# Gender in Facial Representations: A Contrast-Based Study of Adaptation within and between the Sexes

**DOI:** 10.1371/journal.pone.0016251

**Published:** 2011-01-18

**Authors:** Ipek Oruç, Xiaoyue M. Guo, Jason J. S. Barton

**Affiliations:** 1 Department of Ophthalmology and Visual Sciences, University of British Columbia, Vancouver, Canada; 2 Department of Medicine (Neurology), University of British Columbia, Vancouver, Canada; 3 Department of Psychology, University of British Columbia, Vancouver, Canada; 4 Neuroscience Department, Wellesley College, Wellesley, Massachusetts, United States of America; Rand, United States of America

## Abstract

Face aftereffects are proving to be an effective means of examining the properties of face-specific processes in the human visual system. We examined the role of gender in the neural representation of faces using a contrast-based adaptation method. If faces of different genders share the same representational face space, then adaptation to a face of one gender should affect both same- and different-gender faces. Further, if these aftereffects differ in magnitude, this may indicate distinct gender-related factors in the organization of this face space. To control for a potential confound between physical similarity and gender, we used a Bayesian ideal observer and human discrimination data to construct a stimulus set in which pairs of different-gender faces were equally dissimilar as same-gender pairs. We found that the recognition of both same-gender and different-gender faces was suppressed following a brief exposure of 100ms. Moreover, recognition was more suppressed for test faces of a different-gender than those of the same-gender as the adaptor, despite the equivalence in physical and psychophysical similarity. Our results suggest that male and female faces likely occupy the same face space, allowing transfer of aftereffects between the genders, but that there are special properties that emerge along gender-defining dimensions of this space.

## Introduction

Adaptation aftereffects are changes in the perception of a stimulus following exposure to another. Aftereffects are ubiquitous in the visual system, commonly found for many visual dimensions, such as luminance, contrast, spatial frequency, orientation and motion [1], [2], [3], [4], [5], [6], [7] among others. One key aspect of adaptation aftereffects is their selectivity, in that the size of the aftereffect is modulated by the similarity between adapting stimuli and test stimuli. This selectivity is thought to reflect the tuning of the mechanisms that encode these stimuli [8]. According to this view, perception is based on a population of units, each tuned to a limited range of values for a stimulus property (e.g., orientation, direction of motion, spatial frequency). Adapting to a specific value of a stimulus property (e.g., high spatial frequency), affects only those units that respond to that value, and leaves others unaffected. Thus, adaptation has been successfully used as a psychophysical tool to infer the tuning properties of various mechanisms underlying the perception of basic visual properties such as orientation, spatial frequency and direction of motion [1], [9], [10], [11].

Aftereffects also occur for more complex stimuli, such as faces. For example, adapting to a male face causes a gender-neutral face to appear more female [12]. Similar aftereffects have been shown for many different facial attributes, such as identity, ethnicity, expression, and viewpoint [12], [13], [14], [15]. Faces are higher-level stimuli and less is known about their neural encoding compared to our understanding of lower-level “channels” and their tuning properties. The systematic examination of how face aftereffects are modulated by various facial attributes offers a means to explore how faces are represented in the human visual system.

A commonly used metaphor for the organization of face representations is the concept of a ‘*face space*’ [16], [17], where faces are encoded along multiple physical dimensions. Precisely what physical dimensions are represented in human face space is not yet clear; nevertheless, it is these attributes that we use to discriminate one face from another. The concept of face space has been used successfully to explain various effects of ethnicity in face processing, as due to denser representation for faces of an ethnic group not frequently encountered by the observer and therefore, greater distance between faces of one's own race than between faces of the *other race* [18], [19].

How gender is reflected in the organization of face representations has also been a subject of interest and studied using adaptation techniques. Studies of gender-contingent aftereffects and cross-gender transfer of adaptation have yielded a variety of opinions. Some have suggested that male and female faces constitute distinct populations that are functionally independent and show little interaction [20], which others have interpreted as possibly suggesting separate face spaces for males and females [21]. Such a strong version of gender-selectivity would have some difficulty accounting for simple gender aftereffects, in which adapting to e.g. a male face causes a gender-neutral face to appear female [12], and would predict both lack of cross-gender transfer of adaptation, as well as minimal if any influence of the properties of different-gender faces in gender-contingent aftereffects. Others have suggested that male and female faces likely occupy the same face space, largely on the basis that cross-gender transfer of adaptation does occur, but with both gender-selective dimensions and dimensions common to both genders [21].

If male and female faces are represented in the same face space, which the best evidence tends to support [14], [21], [22], it is also possible to ask a slightly different question: do the dimensions that define gender differ in some way from other dimensions in face space? (Note that the concept of ‘gender-defining dimensions’ differs from that of the ‘gender-selective dimensions’ proposed elsewhere [21]. A gender-selective dimension is a dimension along which the faces of one gender vary but those of the other gender do not. A gender-defining dimension is one along which the faces of both genders vary, but in such a way that those of one gender tend to have different values than those of the other, permitting this dimension to contribute to the discrimination of female from male faces.)

Evidence that gender dimensions have a special status could come from adaptation studies showing that aftereffects differ according to whether the adaptor and test faces share the same gender or not. However, if differences are found, it is possible that these could simply reflect the fact that, for a given stimulus set, the stimulus faces of a different gender may be more dissimilar to the test faces than the stimulus faces of the same gender and therefore are located further away in face space from the test faces. However, as others have remarked [21], male and female faces have a high degree of similarity. Therefore it should be possible to select faces for an experimental set in such a way that the physical similarity between different-gender faces is equivalent to that between same-gender faces. If gender influences are found in adaptation with a stimulus set that controls for similarity, this would be more definitive evidence of a gender-specific effect in face-space.

Most previous studies of gender aftereffects have examined *perceptual bias* aftereffects, in that they report on relative shifts of perception along a continuum between two values of a facial property, to one of which the subject is adapted. Ideally, to quantify effects in face space it would be helpful to have a reported metric that is orthogonal and therefore not relative to the facial dimension being adapted. In this study we use a novel face adaptation paradigm first introduced in Oruc & Barton [23] [also see], [ 24,25] that measures changes in recognition contrast thresholds as a result of face adaptation. In more traditional examples of visual adaptation (e.g. orientation) the impact of adaptation on the underlying neural mechanisms has commonly been observed in two main phenomena: selective changes in the sensitivity to the adapting stimulus, and perceptual bias aftereffects [e.g., 9]. Thus in orientation adaptation, prolonged exposure to one specific orientation causes both a selective loss of sensitivity at this orientation, and also a bias towards perceiving a nearby orientation as tilted in the opposite direction, e.g., after adapting to a counterclockwise tilt of 10°, vertically oriented stimuli appear to have a clockwise tilt [4], [11], [26], [27]. These two phenomena are thought to be mediated by a common process in which the response of the adapted unit is selectively suppressed, resulting in the sensitivity loss that in turn brings about an imbalance in the population response to a similar stimulus and thus a shifted or biased percept [3], [28]. While many examples of perceptual bias aftereffects of face adaptation have been reported in the last decade [12], [13], [14], [15], [29], [30], studies on how face adaptation affects sensitivity to faces have generally been lacking.

In Oruc & Barton [23] we measured changes in contrast sensitivity for recognizing a face for a wide range of adapting durations (10ms–6400ms). These results showed that face adaptation involves multiple complex effects. First, as expected on the basis of hypothesized suppression of adapted representations, adaptation decreased sensitivity for recognizing the same face: however, this was found only for adapting durations longer than 500ms. At shorter durations sensitivity actually improved, indicating facilitation and thus imparting a complex non-monotonic pattern to the temporal dynamic of adaptation. Second, adaptation increased recognition thresholds for “different” faces, a result not predicted by simple models based on suppression of adapted representations alone, but which is suggested by more complex views that incorporate a degree of lateral inhibition [31]. In Oruc & Barton [23] we presented modeling results and discussed various implications of this pattern of aftereffects on our understanding of face recognition. In the present study, we chose an adapting duration to test for effects of gender, with the goal of maximizing the ability to detect differences between same- and difference-face aftereffects, preferably by showing facilitation for the former but suppression for the latter.

**Figure 1:**
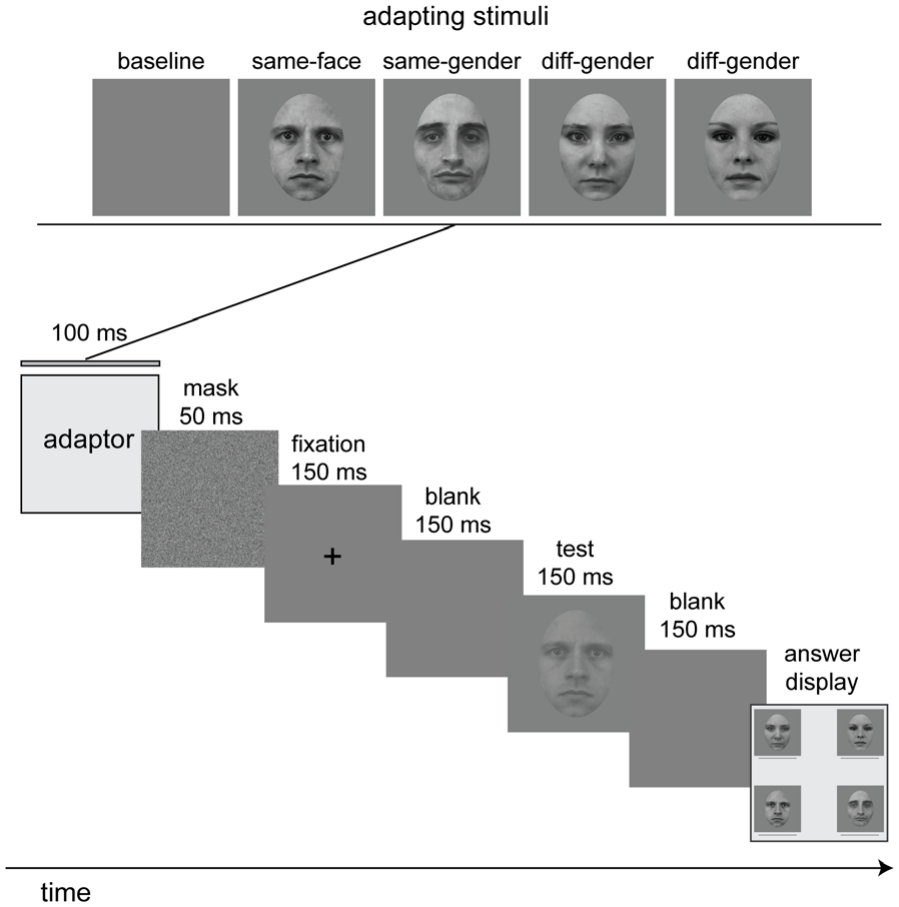
Illustration of a typical trial. Each trial starts with a 100-ms adapting period during which either one of four faces (two females and two males) or a blank stimulus is shown. Following this is a 50-ms noise mask, a 150-ms fixation cross, and a 150-ms blank. After this a test face is shown for 150 ms. The test face is randomly chosen from the same set of four faces that were used as adapting stimuli. The task of the subject is to indicate which one of the four test faces they saw by choosing it from a choice display that remained until the subject entered their response. Contrast thresholds were measured for recognizing each face in a 4-alternative forced-choice (4-AFC) paradigm. A psychophysical staircase controls the contrast of the test face at each trial to estimate thresholds for 82% accuracy. The adapting faces are shown at a fixed rms contrast of 60%. The 20 adapting/test stimulus pairs (five adapt×four test) were further classified into three main conditions: (1) *the same-face condition*, where the test and adapting faces were the same, (2) *the same-gender condition*, where they were different faces of the same gender, (3) *the different-gender condition*, where they were different faces of different genders, in addition to *the baseline condition*, where the adapting stimulus was a blank.

**Figure 2:**
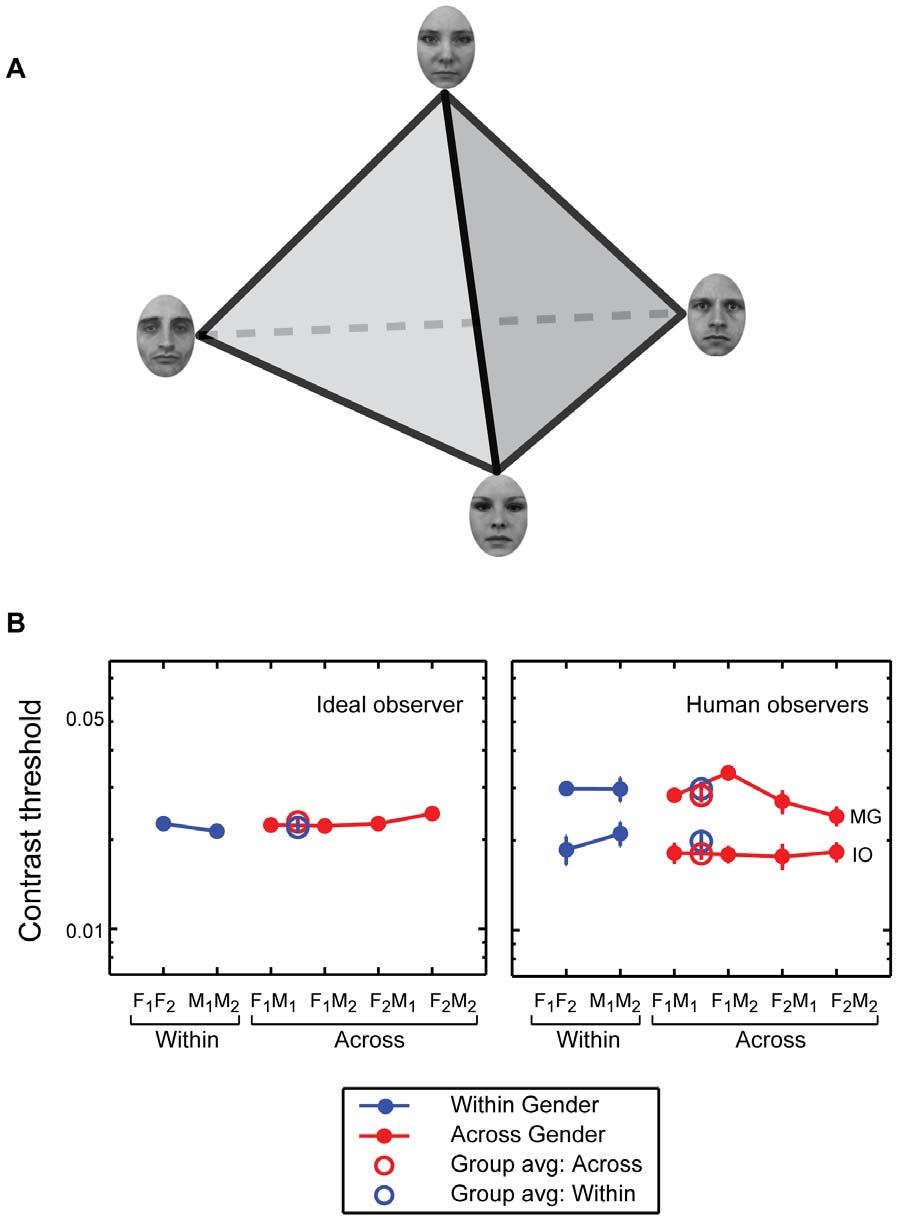
Selection of face stimuli. (A) Our stimulus set contains four faces, two male and two female. In general, the physical dissimilarity between different-gender face pairs would on average be larger than that between same-gender pairs, because a male and a female face differ in those attributes that determine gender in addition to all the other ways in which faces can vary. Therefore, in a randomly selected stimulus set, gender and physical similarity are potentially confounded. To prevent this confound we set out to assemble a set of four faces in which physical dissimilarity between all pairs are equivalent. If the dimensions of the representational space of faces were known, this would simply be a matter of selecting four pair-wise equidistant points in this space. An illustration of such an arrangement is shown. (B) Although dimensions of face space are unknown it is generally accepted that they are those qualities that enable discrimination. In other words, the more similar faces are to each other, the closer they are in face space and the harder it is to discriminate between them. We used a Bayesian ideal observer in a two-alternative forced-choice (2-AFC) task to measure discrimination thresholds between all pairs of faces for a large number of candidate stimulus sets. The ideal observer discrimination contrast thresholds are shown on the left for the best choice that provided the closest approximation to equal discrimination thresholds across all six pairings (a set of two males and two females yield two same-gender and four different-gender pairs). Importantly, for this set of stimuli, discriminating the same-gender face pairs (blue) was as easy as discriminating the different-gender face pairs (red). This was verified by similar results obtained from two human observers (shown on the right).

We used recognition contrast thresholds to determine first, if aftereffects show cross-gender transfer, and second, if these aftereffects differ when the test stimulus has a different gender than the adapting stimulus. Our stimulus set was composed of two male and two female faces, all to be used both as adapting and test faces, so that in any given trial the test face could be the same as the adaptor, a different face of the same gender, or a different face of the other gender (see Figure 1). We chose the particular faces with a selection process that controlled for similarity as measured physically by an ideal observer technique and verified psychophysically with discrimination experiments in human observers (Figure 2). If gender effects in adaptation merely reflect the fact that, on average, faces of a different gender are located further away in face-space than faces of the same gender, then aftereffects should be similar in same-gender and different-gender conditions once we have controlled for similarity. On the other hand, if there is something distinct about the dimensions that determine gender, then differences in adaptation effects should still be evident despite the fact that same-gender and different-gender face pairs are matched for degree of similarity.

## Materials and Methods

### Ethics Statement

The protocol was approved by the review boards of the University of British Columbia and Vancouver Hospital, and written informed consent was obtained in accordance with the principles in the Declaration of Helsinki.

### Subjects

There were nine subjects (five females, ages 18–35) with normal or corrected vision. All but two of the subjects were naïve with respect to the purposes of the experiment.

### Experimental apparatus

Subjects were seated 99 cm from the display screen in an otherwise dark room. The stimuli were presented on a SONY Trinitron 17-in GDM-G500 monitor at 1024×768 resolution and 100Hz refresh rate. A Cambridge Research Systems (CRS) VSG 2/3 video card was used to display stimuli via the CRS VSG toolbox for Matlab and the Psychophysics Toolbox [32], [33]. The display was gamma-corrected using an OptiCAL photometer (Model OP200-E) and software by CRS. Mean luminance was 35 cd/m^2^.

### Stimuli

Two female and two male faces with neutral expression from the Karolinska Database of Emotional Faces [34] were used as stimuli (see Figure 1). Selection of these stimuli was based on two criteria: gender ratings and discriminability.

### Gender ratings

28 male and 33 female faces in the database were first converted to grayscale using Adobe Photoshop (www.adobe.com). An oval mask that was 283×400 pixels in size was superimposed onto the face images. 15 volunteers were asked to rate the faces in terms of their *femaleness* or *maleness* on an 11 point scale, 0 and 10 representing the highest maleness and femaleness scores, respectively. Raters viewed and scored faces in a random order. The average rating for the female faces was 7.1±1.5 (mean ± sd), and the average rating for the male faces was 2.2±1.3. Six female faces with the top femaleness scores (ranging between 8.2–8.7), and 6 male faces with the top maleness scores (ranging between 1.1–1.4) were selected as candidate stimuli. The 12 candidate faces were then aligned horizontally and vertically with respect to the oval mask such that the tip of the nose and pupils were level across all faces. The luminance values inside the oval mask were normalized for each face such that mean luminance was equal to half maximum luminance (i.e., 35 cd/m^2^), and the root-mean-squared (rms) contrast (defined as the standard deviation of luminance divided by mean luminance) was set to 1. Luminance outside the oval mask was set to half the maximum luminance. At this point, the faces that contained trivially distinguishing marks (such as hair visible inside the oval mask, moles, etc.) were eliminated which resulted in the exclusion of 3 of the candidate male faces. The remaining set of 9 faces (6 female, 3 male) were submitted to a discriminability analysis using a Bayesian ideal observer simulation as follows.

### Discriminability

To avoid confounding gender differences with physical similarity, we aimed to create a stimulus set (comprising 2 female and 2 male faces) where pair-wise similarities between all pairs of faces were equal (see Figure 2A for an illustration). This is equivalent to picking four pair-wise equidistant points in face space. How do we accomplish this? If we knew what the dimensions and the distance metric of face space were then it would simply be a matter of computing pair-wise distances across many pairs of faces and finding a set of four (two males and two females) where all pair-wise distances (a total of six distances given by four choose two) are equal. Lacking this information we used an indirect way to measure distances in face space. While the dimensions of face space are unknown, it is commonly agreed that they are those attributes that enable discrimination of faces. In this respect, discriminability of faces provides a distance measure. We measured discrimination contrast thresholds for a Bayesian ideal observer and two human observers, and used these results to guide the selection of our stimuli (Figure 2).

A total of 45 distinct sets of four faces with two faces from each gender can be constructed using the 6 female and 3 male candidate faces obtained. To ascertain the suitable stimulus sets among these that meet the condition of equal discriminability between all pairs, i.e., pairs of same-gender faces equally distinguishable as pairs of different-gender faces, we submitted all 45 potential stimulus sets to an ideal observer analysis [35].

Each potential stimulus set contained 2 female and 2 male faces. This gave 6 pair-wise comparisons within a given stimulus set, two of which were same-gender, and the remaining four were different-gender pairs. A separate discrimination contrast threshold was measured for each pair of faces in a two-alternative forced-choice (2-AFC) paradigm. At each trial one of the two faces, F_i_, i = 1,2, was chosen at random as the test face and shown at variable contrast with added white noise of fixed variance. The test face contrast (controlled by a staircase procedure), the variance of the Gaussian noise (fixed throughout), and the prior probability of either face being presented (equal), were known to the ideal observer. The Bayesian ideal observer based its response on maximum posterior probability, which was equivalent to a minimum distance rule given equal prior probability for the two test faces and our use of Gaussian white noise [36] given by 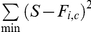, where S is the noisy stimulus, and F*_i,c_* is the i^th^ face template at contrast *c*. Further details of this model can be found in Fox, Oruc, & Barton [37]. The contrast of the test face was controlled by a 40-trial staircase that estimated threshold at 82% accuracy. The ideal observer's contrast threshold for each pair of faces was measured independently 200 times, and the average is reported.

This procedure was repeated for each of the 45 potential stimulus sets to determine the most suitable candidate set of four faces. Note that the specific values of the ideal observer's thresholds were arbitrary as they depend on the noise variance. Rather, what we are looking for is that the 6 thresholds measured for all face pairs in a candidate stimulus set be approximately equal. Upon visual inspection of the ideal observer results for all 45 potential stimulus sets, we selected the set shown in Figure 1. In Figure 2B, left panel, discrimination thresholds of the ideal observer for the six pairs of faces are plotted, in blue for same-gender pairs and in red for different gender pairs. The data plot is quite flat, indicating that all face pairs in this stimulus set were approximately equally discriminable. Most importantly, the same-gender thresholds (blue) were not higher than different-gender thresholds (red) indicating that the similarity among same-gender faces was equivalent to the similarity among different-gender faces.

After using these ideal observer data to select the candidate stimulus set, we then tested two human observers on this stimulus set, to confirm that human perceptual discriminability follows that of physical discriminability with the ideal observer. There was substantial agreement between the human and ideal observer results (Figure 2B, right panel), indicating that both physical and psychophysical similarity between different-gender face pairs was not significantly different than that of the same-gender face pairs, in the stimulus set shown in Figure 1.

Finally, to make sure that our face images, which were equated for pair-wise discriminability, do not retain other distinctions that confer differences in the *appearance* of female and male faces, we also collected similarity ratings. We asked 15 naïve volunteers to rate the similarity of all six face pairs (two same-gender, four different-gender) on a 11-point scale, 0 and 10 representing the lowest and highest similarity, respectively. A Tukey-Kramer multiple comparison test showed no significant differences in the ratings obtained for the six pairs. In addition, a linear contrast (2×same – different) showed no significant difference (p>0.5) between same-gender versus different-gender ratings (3.73 versus 3.54). Thus, the similarity ratings are in line with our discriminability results, eliminating perceived appearance as a potential confound.

### Procedure

We measured recognition contrast thresholds in a four-alternative forced-choice (4-AFC) paradigm and examined how thresholds were impacted as a result of adapting to a face. Each trial started with a 100-ms adaptation to one of four faces (two males and two females), or a blank (see Figure 1). Previously Oruc & Barton [23] have shown that a 100-ms exposure to the adapting face lowers thresholds in the same-face condition below that of baseline (threshold change ratio = 0.67), but elevates thresholds in the different-face condition above baseline (threshold change ratio = 1.21). We chose this adapting duration to take advantage of the cost of a nearly two-fold increase in recognition thresholds associated with adapting to a different face compared to adapting to the same face. The adapting stimulus was followed by a 50-ms white noise mask, a 150-ms fixation period, and a 150-ms blank. Then the test stimulus, one of four faces chosen at random, was shown at low contrast for 150 ms. After a 150-ms blank, an answer display showing all four faces was presented until the subject indicated which one of the four faces they saw by a key-press. Auditory feedback was provided: a single click for a correct response and a double-click for an incorrect response. The contrast threshold for each of 20 adapt-test pairs (five adaptors×four test faces) were measured separately via 20 randomly interleaved staircases each of which ran for a fixed length of 40 trials.

To minimize the contribution of aftereffects from lower-level image properties in our face adaptation paradigm, we incorporated a size and location mismatch between the adapting and test stimuli. The adapting face was presented centrally at fixation with a fixed rms contrast of 0.6. The test faces were 10% smaller in size and were presented 1° left or right of fixation determined randomly at each trial. Since the subjects did not know whether the test face would be displayed on the left or right at any given trial, they were instructed to fixate in the center. The duration of the test face was too short (150 ms) for the subjects to make a foveating saccade and therefore the test faces were viewed slightly peripherally relative to the adapting stimulus. The contrast of the test faces were determined at each trial by a psychophysical staircase implemented in the Psychophysics Toolbox [32], [33] based on the Quest procedure [38].

To familiarize the subjects to the 4-AFC task and the four faces used in the experiment, a short training session was provided prior to the adaptation experiment. During the training, subjects performed the 4-AFC recognition task (without any adaptation period) for four blocks of 40-trials each, or until their performance stabilized determined based on visual inspection of their thresholds in each block.

### Data analysis

There were three main conditions: (1) *same-face*, where the adapting and test faces were the same, (2) *same-gender*, where the test face was a different face of the same gender, and (3) *different-gender*, where the test face was a different face of the different gender, in addition to a *baseline* condition, where the adapting stimulus was a blank. A *threshold change ratio* was computed by dividing the contrast threshold measured for a given adapting condition by that of the corresponding baseline condition (i.e. for that particular test face). If adaptation does not affect performance, then the threshold change ratio would be 1. Values above 1 represent impairment of performance (i.e., threshold elevation), and values below 1 represent facilitation (i.e., threshold reduction). For each subject threshold change ratios for the three main conditions were given by geometric averages of the corresponding test-adapt face pairs. Group data was in turn obtained by taking geometric averages across all subjects.

Threshold change ratio data were then submitted to a Kruskal-Wallis one-way ANOVA to test for a main effect of condition (same-face, same-gender, and different-gender). This was followed by pair-wise comparisons between the three main conditions, as well as between each condition and the baseline using Wilcoxon signed-rank tests. Error bars for the group data were obtained by a non-parametric bootstrap simulation [39], in which the individual data ware re-sampled with replacement a large number of times and analyzed the same way as the experimental data. 68% confidence intervals were then obtained by sorting the resulting data set and selecting the upper and lower 16^th^ percentile values separately for each condition.

## Results

**Figure 3:**
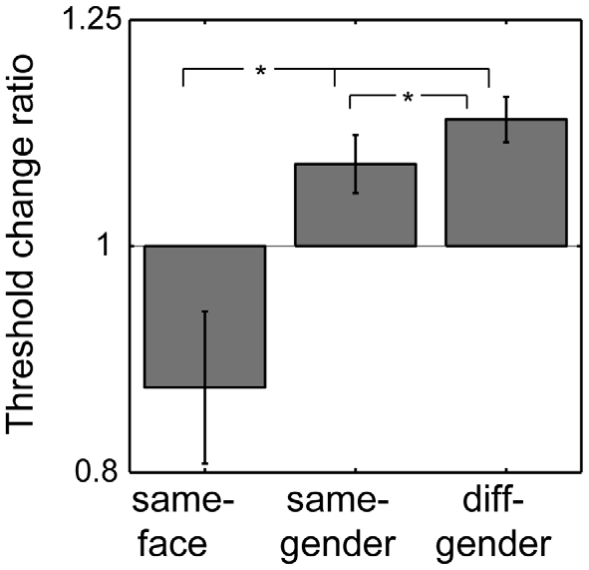
Experimental results. Threshold change ratios for the three main conditions are computed by dividing the contrast threshold in each case with the corresponding baseline threshold, such that a value of one indicates no effect of adaptation. Values below one represent lowered thresholds, i.e., a facilitation effect, and values above one represent elevated thresholds, i.e., a suppression effect. Geometric averages across nine subjects are shown. Adapting to the same face as the test face lowered thresholds below baseline performance, and adapting to a different face elevated thresholds. Most importantly, adapting to a different face elevated thresholds even more if it also differed in gender from the test face. Stars represent significant pair-wise differences. Errorbars are 68% bootstrap confidence intervals.

Contrast thresholds for recognizing faces in a four-alternative forced-choice (4-AFC) task were measured following exposure to the same face (the *same-face* condition), a different face of the same gender (the *same-gender* condition), and a different face of the other gender (the *different-gender* condition). Threshold change ratios were computed by dividing the contrast thresholds in each condition by the baseline threshold determined by using a blank adaptor. Figure 3 shows threshold change ratios plotted for the three main conditions. There was a significant main effect of condition (p<0.01). Adapting to the same face showed a trend to decreased thresholds below baseline (p = 0.07), while adapting to a different face of either gender significantly increased thresholds above baseline (both p's<0.02). All pair-wise comparisons between the three main conditions were significant, with the *same-face* condition significantly lower than both the *same-gender* and the *different-gender* conditions (both p's<0.02), and, most importantly, the *same-gender* condition significantly lower than the *different-gender* condition (p<0.05).

## Discussion

We showed previously that at brief adapting durations an adapting face lowers contrast recognition thresholds for that face but elevates thresholds for other faces, compared to baseline performance [23], [24], [25]. The results of the current experiment replicate this pattern. In addition, our results showed two important findings regarding adaptation effects in the different-gender condition. The first is that there was an aftereffect resembling that in the same-gender condition, consistent with cross-gender transfer of adaptation. The second is that the congruency of gender did influence adaptation, with greater elevation of thresholds occurring in the different-gender than the same-gender condition, even though we had controlled for perceptual similarity.

The finding of cross-gender transfer of adaptation is consistent with a number of previous reports that showed either complete cross-gender transfer for expression [14] and shape aftereffects [21], or partial cross-gender transfer of ethnicity aftereffects [22]. These contrast with another report that variations in the degree of ‘average-ness’ or sexual dimorphism did not generate cross-gender aftereffects in ratings of attractiveness [20]. This discrepancy is likely due to the differences in the properties being adapted in each study. While the structural properties underlying expression, shape and ethnicity are likely similar for both female and male faces, those determining attractiveness differ at least partly between male and female faces [40]. For example, the role of sexual dimorphism may be not only opposite but also asymmetric, with highly feminized faces being most attractive in female faces, but average or only moderately masculinized faces being preferred for male faces. The complex differences in what makes a male versus a female face attractive complicate the interpretation of the results of Little et al. [20]. If the dimensions in face space that generate attractiveness for male faces differ from those for female faces, this alone can account for minimal cross-gender transfer of adaptation for attractiveness ratings. This would imply that such data cannot be taken as evidence of separate neural populations for male and female faces.

Another class of finding used to support the possibility of gender-selective mechanisms or distinct populations is that of gender-contingent aftereffects. This has been shown for inter-ocular distance [20], facial contraction/expansion [21], ethnicity [22], [30] and perceived normality of facial structure [41]. For example, viewing both males with close-set eyes and females with wide-set eyes in the same adapting period leads to a perception that male test images with more wide-set eyes and female test images with more close-set eyes are more normal in appearance [20]. As has been pointed out by Ng et al. [22], completely separate representations for each gender would predict gender-contingent aftereffects as large as same-gender aftereffects when viewing a single face or only faces of one gender, because the presence or absence of the face of the other gender would be irrelevant. However, in those experiments that measured both gender-contingent and same-gender aftereffects, the gender-contingent aftereffects have been weaker than same-gender aftereffects [22].

**Figure 4:**
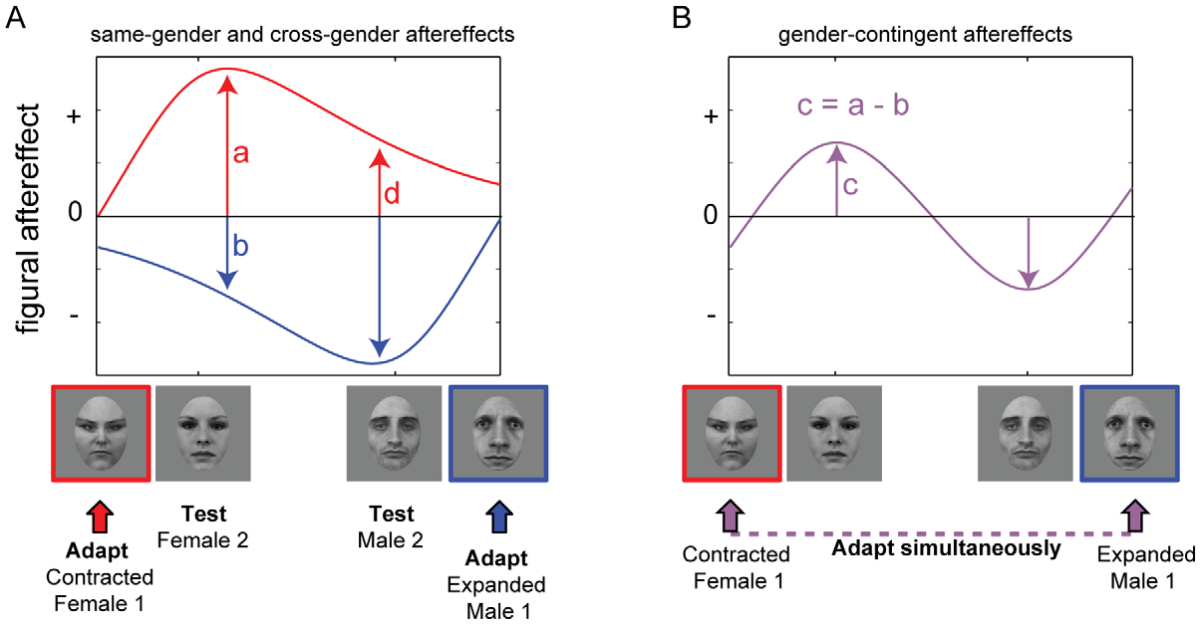
Hypothetical model predictions for magnitude of figural aftereffects in same-gender, cross-gender and gender-contingent conditions based on proximity in face space. (A) Effects of adaptation are dependent on the similarity between the adapting and test stimuli. Perceptual aftereffects peak at a neighboring location, then gradually fall off as the test stimuli become more dissimilar. For example, the effect of adapting to a contracted female face will have greater impact on a female test face (red curve, ‘a’) than a male test face (red curve, ‘d’) simply due to greater similarity between faces of the same gender compared to faces of different genders. The result is cross-gender transfer of aftereffects (‘d’) that is less than the aftereffect for the same-face (‘a’). The same logic applies to the effects of adapting to an expanded male face (blue curve). (B) Gender-contingent aftereffects are obtained by simultaneously adapting to a male and a female face with opposing figural distortions. The fact that contingent aftereffects are usually found to be smaller in magnitude than same-gender aftereffects are predicted by an additive effect of the simultaneous adaptation: Adapting to a contracted female face generates a large perceptual bias on a female test face (red curve, ‘a’), which is offset by a smaller but opposite bias caused by adapting to an expanded male face (blue curve, ‘b’). Thus the contingent aftereffect magnitude ‘c’ will be equivalent to the same-gender aftereffect ‘*a*’ reduced by the cross-gender aftereffect ‘b’.

The fact that both cross-gender adaptation transfer occurs and that gender-contingent aftereffects exist has led to proposals that multiple mechanisms are involved – “common and sex-selective mechanisms” [21] or “singly and jointly tuned mechanisms” [22]. However, it is also possible, and perhaps more parsimonious, to explain these effects as naturally arising from partially overlapping distributions in a single face space of the representations of male and female faces, if aftereffects have some selectivity for the region of face space in which they are evoked (Figure 4). The fact that male and female faces are located in the same face space accounts for cross-gender transfer of adaptation; the fact that they occupy slightly different regions of face space can account for smaller aftereffects when gender is incongruent between test and adaptor [22]. Larger aftereffects in same-gender than in cross-gender conditions will generate gender-contingent aftereffects, though these will be weaker than same-gender aftereffects, since gender-contingent effects emerge from a balance between the same-gender and cross-gender effects in these paradigms.

So far then, the results of previous studies can be explained by spatially separated but not independent distributions of female and male face representations in a single face space. The spatial separation is not controversial, as our ability to distinguish male from female faces indicates that they must differ along some dimensions in face space. However, if faces of different genders occupy differing portions of a single face space, then it becomes important to determine whether gender-related effects in specific experiments can be accounted for simply by greater separation in face space between stimuli differing in gender than between stimuli of the same gender. If so, this would imply that there is no special status derived from the category of gender, vis à vis other sources of structural variation in faces. Few studies to date have attempted to control for face similarity. One study of gender-contingent aftereffects used a prototype-based transformation method to create pairs of female/male faces and pairs of female/hyper-female faces, in which the physical image differences within the female/male pair were linearly similar to those within the female/hyper-female pair [41]. This study reported contingent aftereffects only when male and female faces were paired, not when female and hyper-female faces were paired. This suggests a categorical effect of gender beyond the effects of physical similarity. However, it is not know whether the metrics of face space follow the linear distance metrics used to make physically equivalent stimuli in this experiment, and the authors acknowledged that controlling for physical difference in a two-dimensional image does not guarantee equivalent perceptual differences between faces.

Nevertheless, our second finding, that same-gender aftereffects differ from different-gender aftereffects, also supports a conclusion that the effects of gender are not reducible to the psychophysical separation of male and female faces in this face space. We used a different approach to control similarity in same-gender and different-gender pairs than that used by [41], with measures that equated both physical and perceptual differences in the stimuli. In the absence of knowledge of the dimensions that define face space, we, like others in the past [18], [42], [43], used measures of distinctiveness or similarity to index the distance between representations in face space. Our ideal observer and human observer data show that, in both physical and psychophysical terms, the faces in our stimulus set were as similar to the faces of the opposite gender in the set as much as they were to faces of the same gender. Despite this, adaptation increased thresholds more for different-gender faces than for same-gender faces. This suggests that there is something anomalous occurring along gender-relatedz dimensions in face space. The nature of this anomaly is not yet clear, but there are several potential, inter-related explanations upon which one could speculate. One may be that distances in face-space do not reflect merely similarity, but include factors that distort or increase perceptual distances along gender-defining dimensions. How this would occur is not clear. However, one possible physiological basis for such an effect might be that there are differences in lateral connectivity between representations along gender-defining dimensions compared to other dimensions, so that the spread of adaptation-related activity differs when gender changes. This would be in line with the suggestion that adaptation suppresses the representation of other faces through lateral inhibition [23], [31], causing the elevated contrast recognition thresholds we observe in the different-face conditions in this and other similar experiments [23], [24], [25]. Last, from a cognitive point of view, it may be that this anomaly is introduced by the crossing of a categorical boundary, which during adaptation confers an added degree of suppression upon representations on the other side of the boundary. A categorical effect would also be supported by the finding of [41] that, with hyper-female, female and male faces all modified along the same physical dimensions, contingent aftereffects arise only when the paired faces differ in gender. It is also consistent with previous work using classic paradigms that show better discrimination across categorical boundaries then within categories, which conclude that such effects can be found for face gender [44], [45]. just as they can be found for face identity [46] and face expression [47], [48].

Of course, these different levels of explanation are not mutually exclusive. The physiological foundations of the categorical effect in high-level vision remain elusive: others have pointed out that the “mechanisms by which faces are perceived categorically have yet to be adequately accounted for by any theoretical approach” [46]. Some suggest that part of the categorical effect reflects a ‘between-categorical separation effect’, in which acquired distinctiveness reflects “an increase in perceptual sensitivity to differences that are relevant for a categorization” [44]. As is evident, this corresponds closely to the first proposal of distortion or increase in perceptual distances along gender dimensions and it may be that increased lateral inhibition along gender dimensions could be one means of achieving this in physiological terms.

In conclusion, while our results and those of others best support overlapping or adjacent distributions of male and female face representations within the same face space, our findings go beyond those of prior studies, to provide evidence of additional effects introduced by the category of gender, such that adaptation is associated with even greater suppression of different-gender face representations. Thus, while it is unlikely that male and female faces occupy separate and independent face spaces, gender may nevertheless confer some special status to certain dimensions within face space.
